# Diet and breast cancer in Shanghai and Tianjin, China.

**DOI:** 10.1038/bjc.1995.263

**Published:** 1995-06

**Authors:** J. M. Yuan, Q. S. Wang, R. K. Ross, B. E. Henderson, M. C. Yu

**Affiliations:** Shanghai Cancer Institute, People's Republic of China.

## Abstract

Various aspects of adult diet have been linked to breast cancer development. These include intake of fat (risk factor), and intake of fibre, soy protein and vitamins A, C and E (protective factors). Results of previous studies have been inconsistent. We examined the possible associations between breast cancer and various indices of nutrient and food intake in two Chinese populations who are at relatively low risk for breast cancer (one-fifth the rate in US white women). Two case-control studies of breast cancer were conducted in the cities of Shanghai and Tianjin, China. In Shanghai, 534 women aged 20-69 years with histologically confirmed breast cancer were recruited, whereas in Tianjin 300 women aged 20-55 years with histologically confirmed breast cancer were interviewed. All controls were community controls who were individually matched to the cases by sex and age (case-control ratio = 1:1). All interviews were conducted in person. Findings from the two studies were similar, although the diets in Shanghai and Tianjin were different in many respects. Cases and controls were similar in their consumption of soy protein, measured either in absolute levels or as percentages of total protein. Overall, all components of dietary fat (saturated fat, monounsaturated fat, polyunsaturated fat) showed a modest, non-significant association with breast cancer after adjustment for energy intake and other non-dietary risk factors for breast cancer. Intake of crude fibre, carotene and vitamin C, on the other hand, exhibited strong, statistically significant inverse associations with breast cancer risk. The last three indices were highly correlated, rendering it impossible to disentangle their individual effects; they were closely associated with intake of green vegetables in the two study populations. Vitamin E intake was unrelated to breast cancer risk in Shanghai and Tianjin. In the multivariate logistic regression model which included all non-dietary risk factors for breast cancer and energy intake, Shanghai women in the lowest tertile of crude fibre intake and highest tertile of fat intake had a 2.9-fold increased risk for breast cancer relative to those in the highest tertile of crude fibre intake and lowest tertile of fat intake. The comparable relative risk in Tianjin women was 2.4. Our data indicate a strong protective effect against breast cancer development with intake of foods rich in fibre, vitamin C and carotene. Our results are also compatible with dietary fat having a modest, positive effect on breast cancer risk within the range of exposure experienced by women in China.(ABSTRACT TRUNCATED AT 250 WORDS)


British Journal of Cancer (1995) 71, 1353-1358

© 1995 Stockton Press All rights reserved 0007-0920/95 $12.00

### Diet and breast cancer in Shanghai and Tianjin, China

J-M Yuan[1], Q-S Wang[2], RK Ross[3], BE Henderson[4] and MC Yu[3]

[1]*Shanghai Cancer Institute, Shanghai 200032, People's Republic of China;* [2]*Tianjin Cancer Institute, Tianjin 300060, People's Republic of China;* [3]*Kenneth Norris Jr, Comprehensive Cancer Center, University of Southern California, 1441 Eastlake Avenue, Los Angeles, California 90033-0800, USA;* [4]*The Salk Institute for Biological Studies, La Jolla, California 92037, USA.*

**Summary** Various aspects of adult diet have been linked to breast cancer development. These include intake of fat (risk factor), and intake of fibre, soy protein and vitamins A, C and E (protective factors). Results of previous studies have been inconsistent. We examined the possible associations between breast cancer and various indices of nutrient and food intake in two Chinese populations who are at relatively low risk for breast cancer (one-fifth the rate in US white women). Two case-control studies of breast cancer were conducted in the cities of Shanghai and Tianjin, China. In Shanghai, 534 women aged 20-69 years with histologically confirmed breast cancer were recruited, whereas in Tianjin 300 women aged 20-55 years with histologically confirmed breast cancer were interviewed. All controls were community controls who were individually matched to the cases by sex and age (case-control ratio = 1:1). All interviews were conducted in person. Findings from the two studies were similar, although the diets in Shanghai and Tianjin were different in many respects. Cases and controls were similar in their consumption of soy protein, measured either in absolute levels or as percentages of total protein. Overall, all components of dietary fat (saturated fat, monounsaturated fat, polyunsaturated fat) showed a modest, non-significant association with breast cancer after adjustment for energy intake and other non-dietary risk factors for breast cancer. Intake of crude fibre, carotene and vitamin C, on the other hand, exhibited strong, statistically significant inverse associations with breast cancer risk. The last three indices were highly correlated, rendering it impossible to disentangle their individual effects; they were closely associated with intake of green vegetables in the two study populations. Vitamin E intake was unrelated to breast cancer risk in Shanghai and Tianjin. In the multivariate logistic regression model which included all non-dietary risk factors for breast cancer and energy intake, Shanghai women in the lowest tertile of crude fibre intake and highest tertile of fat intake had a 2.9-fold increased risk for breast cancer relative to those in the highest tertile of crude fibre intake and lowest tertile of fat intake. The comparable relative risk in Tianjin women was 2.4. Our data indicate a strong protective effect against breast cancer development with intake of foods rich in fibre, vitamin C and carotene. Our results are also compatible with dietary fat having a modest, positive effect on breast cancer risk within the range of exposure experienced by women in China. Our study does not support the hypothesis that high intake of soy protein protects against breast cancer.

**Keywords**: breast cancer; dietary fat; dietary fibre; case-control study; China

### Introduction

The search for a dietary component to the aetiology of breast cancer has been the subject of numerous epidemiological studies. The majority of these studies have focused on a possible relationship with dietary fat. Results to date have been equivocal. Eleven of 16 case-control studies in which specific individual estimates of fat intake are available show evidence of a modest, positive association (Howe, 1992). On the other hand, the overall evidence from ten prospective studies, each of which has at least 50 incident cases of breast cancer, does not support such an association (Hunter and Willett, 1993).

A number of case-control and cohort studies have examined the possible associations between breast cancer and dietary intake of fibre and vitamins A, C and E. In general, results of case-control studies demonstrate a reduced risk of breast cancer with high intake of fibre, vitamin C and β-carotene, but no relationship with intake of retinol and vitamin E (Howe *et al.*, 1990; Lee *et al.*, 1991; Zaridze *et al.*, 1991; Hunter and Willett, 1993). The three cohort studies that have investigated these dietary components have produced diverse results. The Canadian National Breast Screening Study found a significant protective effect with high intake of dietary fibre and smaller, statistically non-significant reductions in risk with increasing intake of dietary retinol, β-carotene and vitamin C (Rohan *et al.*, 1993). The US Nurses' Health Study, on the other hand, observed no relationship with dietary fibre or vitamin C, but protective effects associated with high intake of retinol and β-carotene (Willett *et al.*, 1992; Hunter *et al.*, 1993). The New York State Prospective Study (Graham *et al.*, 1992) did not find any associations with dietary fibre or vitamin A, but did note a non-significant, inverse association with vitamin C intake. Vitamin E was unrelated to breast cancer risk in all three cohort studies (Graham *et al.*, 1992; Willett *et al.*, 1992; Hunter *et al.*, 1993; Rohan *et al.*, 1993).

Recently, Lee *et al.* (1991) proposed that high intake of soy protein may protect against breast cancer. The investigators reported decreasing risk of breast cancer with increasing intake of soy protein among premenopausal Chinese women in Singapore, whose diet is relatively high in soy bean products. Soya beans are a rich source of phyto-oestrogens, and it was suggested that sufficient quantities of these plant oestrogens might partly suppress endogenous oestrogenic activity, thereby reducing the risk of breast cancer (Lee *et al.*, 1991).

In the mid-1980s, we initiated two case-control studies of breast cancer in the two largest urban centres of China. One of the goals was to evaluate adult diet, especially dietary fat and certain micronutrients, in relation to breast cancer development in these low-risk populations (the annual age-standardised incidence of female breast cancer in Shanghai and Tianjin is about 19/100 000, one-fifth the comparable rate in US white women). These two studies are well suited to evaluate further the provocative hypothesis that soy bean intake may protect against breast cancer; various soy bean products are an integral part of the diet in Shanghai and Tianjin. We have previously reported the results of non-dietary risk factors from these two studies (Yuan *et al.*, 1988; Wang *et al.*, 1992). This paper presents the dietary findings of the two studies separately and combined.

Correspondence: MC Yu
Received 20 July 1994; revised 2 December 1994; accepted 27 January 1995

### Materials and methods

The recruitment of cases and controls in Shanghai and Tianjin has been described in detail previously (Yuan *et al.*, 1988; Wang *et al.*, 1992). Briefly, eligible cases in Shanghai were all histologically confirmed incident cases of breast cancer diagnosed between 1 June 1984 and 31 May 1985 among female residents of Shanghai aged 20–69 years, identified through the population-based Shanghai Cancer Registry. Ninety-four per cent of eligible patients were interviewed ($n = 534$). In Tianjin, eligible cases were all histologically confirmed incident cases in women aged 20–55 years that were identified by the population-based Tianjin Cancer Registry beginning on 1 January 1985 until 300 cases had been recruited into the study. Sixty-nine per cent of eligible patients were interviewed. The cancer registries in Shanghai and Tianjin have been in operation since 1963 and 1978 respectively. Their inclusion in the Cancer Incidence in Five Continents series published by the International Agency for Research on Cancer (IARC) attests to the high quality and relative completeness of their data.

Controls in both studies were community controls, individually matched to the index case by sex and age (case–control ratio = 1:1), and selected according to a predetermined algorithm as described previously (Yuan *et al.*, 1988; Wang *et al.*, 1992). In Shanghai, the age-matching criterion specified that the case and her matched control had to belong to the same 5 year age group (20–24, 25–29, . . . 65–69), whereas in Tianjin the control was within 1 year of age to the index case. Virtually all controls interviewed in Shanghai and Tianjin were the first control chosen.

All interviews were conducted in the homes of study subjects by a trained interviewer employing a structured questionnaire. The questionnaires used in Shanghai and Tianjin were similar and covered demographic characteristics, height and usual adult weight, use of tobacco and alcohol, medical history, use of exogenous hormones, menstrual and reproductive history, usual adult diet and family history of cancer. The non-diet-related information collected has been described in detail previously (Yuan *et al.*, 1988; Wang *et al.*, 1992). In Shanghai, the usual frequencies and amounts of consumption of 63 food items were obtained during the interview. Intake frequencies were expressed in number of times per day, week, month or year, depending on the subject's choice. The amount of food consumed was given as the market weight (i.e. before cooking and including roughage) of the food eaten per day, week, month or year. Owing to the food rationing system in place in China from 1950 until recently, our study subjects had no difficulty in relating the amounts of their food intake by market weights. This method of obtaining information on intake amount also provided the most direct way of computing nutrient intake since the Chinese Food Composition Tables (Institute of Nutrition and Food Hygiene, Chinese Academy of Preventive Medicine, 1980; Institute of Nutrition and Food Hygiene, Chinese Academy of Preventive Medicine, 1991) list nutrient values per 100 g of the market weight of a given food item. The list of 63 food items asked about in Shanghai is given in the appendix.

In Tianjin, 68 food items were listed on the questionnaire (see appendix for details). For starches (noodles and rice) and cooking oils, each subject was asked to provide information on amounts (market weights) eaten per day or month, whichever time unit was preferable to the subject. For the remaining food items (meats, fish, eggs, legumes, vegetables and fruit), the subjects were asked to provide information only on usual frequencies of intake (expressed in numbers of times per day, week, month or year). In order to establish a standard portion size for each of these foods, 25 control subjects were asked to indicate, using the actual, uncooked foods, the usual amounts they would consume per meal. For each food, the mean weight of all recorded servings was taken as the market weight of the standard portion. We did not ask about use of vitamin supplements in either study location; use of these products was extremely rare in China during the period of our studies.

For each study subject, we computed usual daily intake of 22 nutrients by combining information obtained from interview with nutrient values from the Chinese Food Composition Tables (Institute of Nutrition and Food Hygiene, Chinese Academy of Preventive Medicine, 1980; Institute of Nutrition and Food Hygiene, Chinese Academy of Preventive Medicine, 1991). Although crude fibre represents only a portion of total dietary fibre, the Chinese Food Composition Tables do not provide values on dietary fibre, and the only available surrogate for fibre intake is crude fibre, which we used for all analyses related to fibre. Cases and controls were compared in terms of their intake levels of various nutrients (as continuous variables and as categorical variables in tertiles or quartiles). Estimates of relative risks and their associated confidence intervals and $P$-values were computed using multivariate conditional logistic regression methods (Breslow and Day, 1980). Possible heterogeneity between studies was always tested whenever the two study data sets were combined in the analysis. Three statistical models have been proposed for simultaneously considering the effects of total calories and a nutrient which contributes to calories (Howe, 1989). Parallel analyses using these three approaches were applied to the various components of energy-generating macronutrients. Results from the different analyses were comparable, and figures shown in this report were taken from the analysis using model 1 as defined in Howe (1989). Cases and controls also were compared according to their intake levels of individual foods, using both parametric ($t$-test) and non-parametric (Wilcoxon signed-rank test) statistical procedures. The two sets of results were comparable, and only the parametric test results are presented in this report. All $P$-values quoted are two-sided.

### Results

Table I presents selected demographic and reproductive characteristics of the study subjects in Shanghai and Tianjin. In

**Table I** Demographic reproductive characteristics of breast cancer cases and controls in Shanghai and Tianjin, China

| | *Shanghai* | | *Tianjin* | |
|---|---|---|---|---|
| | *Cases* | *Controls* | *Cases* | *Controls* |
| Mean age (years) | 50.8 | 50.0 | 43.3 | 43.3 |
| Mean height (cm) | 158.4 | 157.8 | 160.2 | 159.5 |
| Mean weight in reference year[a] (kg) | 52.6 | 51.9 | 59.3 | 58.9 |
| Percentage with college education | 11.4 | 3.2 | 11.7 | 6.7 |
| Mean age at menarche (years) | 14.6 | 15.0 | 14.1 | 14.4 |
| Percentage nulliparous | 12.7 | 8.2 | 11.3 | 9.0 |
| Mean number of full-term births | 2.8 | 3.5 | 2.2 | 2.7 |
| Mean age at first full-term birth | 24.4 | 23.2 | 25.5 | 24.1 |
| Mean duration of nursing (months) | 31.5 | 42.8 | 36.5 | 46.8 |
| Percentage ever used oral contraceptives (OC) | 18.5 | 17.8 | 34.7 | 32.7 |
| Percentage first used OC at age 35 + years | 4.3 | 2.1 | 11.0 | 4.0 |

[a]Reference year = year of cancer diagnosis (for controls, year of interview) minus 2.

both locales, breast cancer cases relative to controls, in general, were more educated, started menstruation earlier, were more likely to be nulliparous and were more likely to use oral contraceptives for the first time after age 35 years. Among the parous subgroups, cases had fewer full-term births, had later age at first full-term birth, and had shorter duration of nursing (see Yuan *et al.*, 1988, and Wang *et al.*, 1992, for details).

Table II presents the percentiles (25th, 50th and 75th) of selected nutritional variables in cases and controls by study location. The relative risks of these nutritional factors in relation to breast cancer are given in Table III. Univariate analyses showed that cases and controls in both locales were similar in intake of total energy, total protein and total carbohydrate, but cases had significantly higher intake of all components of fat (saturated, monounsaturated, polyunsaturated) relative to controls with the exception of polyunsaturated fat, which showed no association with breast cancer in Tianjin. Dietary cholesterol was higher among cases than controls in Shanghai, but this positive association with breast cancer was not evident in Tianjin. Crude fibre intake was significantly lower among cases than controls in both Shanghai and Tianjin, as were intakes of vitamin C and carotene. Intake of soy protein (measured either in absolute levels or as percentages of total protein), retinol or vitamin E was not related to breast cancer in either study location (Table III).

Table III also presents the relative risks of the various nutrients to breast cancer after adjustment for energy intake. Intake of total protein and soy protein remained unrelated to breast cancer risk, while adjustment for energy intake generally strengthened the positive associations noted earlier between breast cancer and the various dietary fat components. Energy adjustment also strengthened the negative associations observed earlier between breast cancer and intake of crude fibre, vitamin C and carotene.

However, the dietary fat–breast cancer associations were attenuated when non-dietary risk factors for breast cancer were controlled for in the analysis. The increases in adjusted relative risks between the highest and lowest quintiles of intake of saturated and unsaturated fatty acids were reduced to about 20–30% and were not statistically significant (Table III).

In contrast, intake of crude fibre, vitamin C and carotene remained significantly and inversely related to breast cancer risk after adjustment for energy intake and other non-dietary confounders (Table III). Intake of these micronutrients was highly correlated in our study populations, being closely associated with vegetable intake. The correlation coefficient ($r$) between vitamin C and crude fibre was 0.89, that between vitamin C and carotene was 0.92 and that between crude fibre and carotene was 0.81. Therefore, these three indices are indistinguishable in terms of their impact on breast cancer risk in Shanghai and Tianjin. Of these three nutrients, crude fibre was the only one that exhibited statistically significant associations with breast cancer risk in both Shanghai and Tianjin. Crude fibre was also the nutrient with the strongest biological plausibility as a direct protective factor for breast cancer (see Discussion below). Therefore, crude fibre intake was chosen as the marker for exposure to either one of these three micronutrients in subsequent analyses.

Tables IV and V present the combined effects of intake of fat and crude fibre on the risk of breast cancer in Shanghai and Tianjin respectively. In Shanghai, the risk-enhancing effect of fat consumption was most evident among women in the lowest tertile of crude fibre intake. Similarly, the protective effect of high intake of crude fibre was most evident among subjects in the highest tertile of fat consumption. Women in the lowest tertile of crude fibre intake and highest tertile of fat intake had the highest risk for breast cancer (Table IV).

In Tianjin, at every level of crude fibre intake, breast cancer risk increased with increasing fat consumption. Similarly, at every level of fat consumption, breast cancer risk decreased with increasing intake of crude fibre. Again, women in the lowest tertile of crude fibre intake and highest tertile of fat intake exhibited the highest level of breast cancer risk (Table V).

We repeated the above analyses separately for pre- and post-menopausal women. Results were similar for the two groups and similar to the overall findings.

In terms of individual food intake, cases relative to controls, both in Shanghai and Tianjin, had a higher intake of pork and cow milk, and lower intake of green vegetables (Table VI). The staple vegetable in Shanghai is bokchoi (a medium green leafy vegetable), whereas in Tianjin the staple vegetable is Chinese cabbage (a pale green vegetable). Among controls in Shanghai, bokchoi consumption accounted for 44% of vitamin C intake. Among controls in Tianjin, 35% of vitamin C intake was derived from Chinese cabbage.

Beef was a rarely consumed food item in both Shanghai and Tianjin. Consumption of chicken and fish was more common, but neither was related to breast cancer risk in either study area. In Shanghai only, cases consumed significantly more eggs than controls.

In both Shanghai and Tianjin, tofu consumption was

**Table II** Percentile (25th, 50th and 75th) nutritional characteristics of breast cancer cases and controls in Shanghai and Tianjin, China

| | *Shanghai (534 case–control pairs)* | | | | | | *Tianjin (300 case–control pairs)* | | | | | |
|---|---|---|---|---|---|---|---|---|---|---|---|---|
| | *Cases* | | | *Controls* | | | *Cases* | | | *Controls* | | |
| *Daily intake of energy nutrients* | $P_{25}$ | $P_{50}$ | $P_{75}$ | $P_{25}$ | $P_{50}$ | $P_{75}$ | $P_{25}$ | $P_{50}$ | $P_{75}$ | $P_{25}$ | $P_{50}$ | $P_{75}$ |
| Total energy (kcal) | 1857 | 2231 | 2628 | 1864 | 2181 | 2623 | 1707 | 1922 | 2179 | 1653 | 1884 | 2183 |
| Total protein (g) | 55.0 | 66.1 | 84.8 | 54.9 | 67.2 | 83.8 | 43.4 | 51.5 | 63.0 | 42.7 | 50.1 | 60.7 |
| Soy protein (g) | 2.4 | 3.5 | 11.1 | 2.4 | 3.5 | 12.6 | 1.2 | 3.5 | 7.5 | 1.2 | 2.8 | 7.1 |
| Soy total protein (%) | 4.0 | 6.4 | 12.9 | 4.3 | 6.8 | 13.2 | 2.5 | 6.6 | 13.2 | 2.3 | 5.5 | 12.8 |
| Total fat (g) | 43.0 | 57.7 | 79.0 | 37.9 | 52.1 | 73.5 | 52.1 | 64.8 | 80.0 | 48.9 | 60.4 | 75.8 |
| Saturated fat (g) | 9.7 | 14.4 | 21.3 | 8.0 | 12.5 | 18.9 | 12.9 | 17.7 | 24.4 | 11.2 | 16.1 | 20.5 |
| Monounsaturated fat (g) | 17.9 | 24.3 | 33.9 | 14.9 | 22.0 | 31.4 | 21.4 | 26.8 | 33.1 | 19.5 | 24.3 | 31.9 |
| Polyunsaturated fat (g) | 11.7 | 15.4 | 20.1 | 10.5 | 14.5 | 19.6 | 14.4 | 17.6 | 20.7 | 14.2 | 17.5 | 20.8 |
| Carbohydrate (g) | 279.0 | 338.9 | 407.5 | 296.5 | 345.4 | 406.6 | 240.8 | 279.4 | 317.2 | 243.2 | 282.5 | 322.6 |
| Cholesterol (mg) | 114.8 | 196.8 | 355.0 | 99.9 | 178.1 | 328.9 | 191.8 | 310.6 | 397.9 | 202.2 | 310.7 | 390.3 |
| Total crude fibre (g) | 3.9 | 4.9 | 6.3 | 4.1 | 5.3 | 6.6 | 2.4 | 2.8 | 3.4 | 2.5 | 3.0 | 3.6 |
| Cereal crude fibre (g) | 1.6 | 2.0 | 2.5 | 1.8 | 2.1 | 2.5 | 1.0 | 1.2 | 1.5 | 1.1 | 1.2 | 1.5 |
| Vegetable fruit crude fibre (g) | 1.8 | 2.6 | 3.9 | 2.0 | 2.9 | 4.4 | 1.2 | 1.6 | 2.0 | 1.2 | 1.6 | 2.2 |
| Vitamin C (mg) | 61.3 | 87.7 | 122.3 | 67.9 | 96.8 | 150.6 | 22.2 | 27.3 | 35.1 | 23.6 | 29.7 | 36.3 |
| Retinol (IU) | 191 | 432 | 805 | 154 | 385 | 799 | 313 | 610 | 759 | 317 | 606 | 691 |
| Carotene (IU) | 2731 | 3992 | 5480 | 3203 | 4518 | 5869 | 750 | 1042 | 1269 | 787 | 1040 | 1271 |
| Vitamin E (mg) | 16.4 | 20.9 | 28.5 | 15.2 | 19.9 | 30.9 | 19.2 | 23.7 | 27.6 | 20.2 | 24.4 | 29.0 |

similar between cases and controls. Consumption of soy milk also showed little difference between cases and controls. Tofu was the major source of soy protein in Shanghai, whereas in Tianjin soy milk was the major source.

Use of cooking oils was similar between cases and controls in both locations. Use of alcohol was uncommon among women in Shanghai and Tianjin, and there was no association with breast cancer in these limited data. Fresh fruits or juices were not readily available and thus were infrequently consumed in both locations.

### Discussion

Our dietary questions were designed to include all common foods in the local diet as well as local foods rich in any of the nutrients of interest. The two questionnaires have not been subjected to formal validation tests (such as comparison to a series of 3 or 7 day food records collected over the 12 months preceding the administration of the standard questionnaires). However, a comparison of the nutritional profile of our control subjects in Shanghai with those from a case–control study of colorectal cancer conducted in neighbouring Zhejiang and employing a detailed, but different, dietary questionnaire revealed a high degree of comparability between the two data sets (Whittemore *et al*., 1990). Thus, there is some assurance that our dietary data are reproducible and correlate with usual adult intake.

**Table IV** Multivariate relative risks[a] for breast cancer in Shanghai by tertiles of intake of total fat and crude fibre

| | *Total fat* First tertile | *Second tertile* | *Third tertile* | |
|---|---|---|---|---|
| Crude fibre | | | | |
| First tertile | 1.06 | 1.40 | 2.92 | 1.81* |
| | (81/99)[b] | (74/63) | (54/15) | (209/177) |
| Second tertile | 0.87 | 1.41 | 1.18 | 1.48* |
| | (36/59) | (67/61) | (70/57) | (173/177) |
| Third tertile | 1.00 | 1.01 | 0.73 | 1.00 |
| | (10/18) | (42/53) | (93/102) | (145/173) |
| | 1.00 | 1.41 | 1.36 | |
| | (127/176) | (183/177) | (217/174) | |

[a]Adjusted for energy intake and non-dietary risk factors in Shanghai as listed under footnote in Table III. [b]Number of cases/number of controls. *Two-sided $P$-value $<0.05$.

**Table V** Multivariate relative risks[a] for breast cancer in Tianjin by tertiles of intake of total fat and crude fibre

| | *Total fat* First tertile | *Second tertile* | *Third tertile* | |
|---|---|---|---|---|
| Crude fibre | | | | |
| First tertile | 2.07 | 2.12 | 2.44 | 2.15* |
| | (47/49)[b] | (46/36) | (26/15) | (119/100) |
| Second tertile | 0.98 | 1.58 | 1.98 | 1.35 |
| | (21/36) | (39/34) | (41/29) | (101/99) |
| Third tertile | 1.00 | 0.80 | 1.52 | 1.00 |
| | (7/15) | (17/28) | (54/56) | (78/99) |
| | 1.00 | 1.18 | 1.66 | |
| | (75/100) | (102/98) | (121/100) | |

[a]Adjusted for energy intake and non-dietary risk factors in Tianjin as listed under footnote in Table III. [b]Number of cases/number of controls. *Two-sided $P$-value $<0.05$.

These data from two major Chinese urban areas do not support the hypothesis that high intake of soy protein protects against breast cancer. Intake of soy protein, in terms of either absolute levels or percentages of total protein or soy bean products, did not differ between breast cancer cases and controls in either Shanghai or Tianjin. Among Chinese Singaporeans, in whom the inverse association between soy protein intake and breast cancer risk was first observed (Lee *et al*., 1991), the median daily intake of soy protein was 2.5 g, or 7% of total protein. Among women in Shanghai and Tianjin, China, the comparable figures are 3.5 g (or 6.8% of total protein) and 2.8 g (or 5.5% of total protein) respectively. Therefore, exposure levels to soy protein among our study populations are similar to those experienced by Chinese Singaporean women.

Our data suggest that, among Chinese women, a diet that is high in pork intake and low in vegetable intake, especially green vegetables, predisposes to breast cancer development. In terms of nutrients, this high-risk dietary profile translates to a modest, positive association with total fat intake, and stronger, negative associations with intake of crude fibrer, vitamin C and carotene. As noted above, our study could not adequately address the separate effects of the last three nutrients owing to the high correlations among them. The correlation coefficient between any two of these three nutrients was at least 0.8.

Recent, prospective studies of breast cancer have cast doubt on earlier observations, based on case–control design, of a positive association between dietary fat and breast cancer (Hunter and Willett, 1993). Our data are compatible with the null hypothesis (none of the dietary fat/breast cancer associations after adjustment for energy intake and confounding factors was statistically significant), but could be interpreted to support a weak, positive association between fat intake and breast cancer risk within the range of exposure studied (ranging from a mean of 15% of total caloric intake among Shanghai women in the lowest quartile category to a mean of 35% among Tianjin women in the highest quartile group). These levels of exposure are considerably lower than those experienced by Western women, who were the subjects of the published cohort results (Hunter and Willett, 1993).

Our data strongly suggest that a diet high in food sources rich in fibre, carotene and vitamin C within the range of exposure studied protects against breast cancer. It has been

**Table VI** Mean daily intake (g) of individual foods among breast cancer cases and controls in Shanghai and Tianjin, China

| | *Shanghai* Cases | *Controls* | *Two-sided P-value* | *Tianjin* Cases | *Controls* | *Two-sided P-value* |
|---|---|---|---|---|---|---|
| Pork | 68.3 | 55.8 | 0.0004 | 39.8 | 36.0 | 0.06 |
| Beef/mutton | 5.4 | 3.9 | 0.10 | 4.3 | 3.5 | 0.24 |
| Poultry | 15.2 | 15.3 | 0.96 | 1.3 | 1.2 | 0.66 |
| Fish | 31.8 | 29.8 | 0.48 | 10.0 | 11.5 | 0.10 |
| Eggs | 24.5 | 21.3 | 0.04 | 36.7 | 37.6 | 0.55 |
| Rice | 373.5 | 386.9 | 0.07 | 124.3 | 121.4 | 0.57 |
| Noodles/bread | 49.6 | 43.7 | 0.12 | 217.2 | 221.4 | 0.51 |
| Medium dark-green vegetables | 164.4 | 189.1 | 0.0001 | 19.4 | 20.0 | 0.37 |
| Light-green vegetables | 99.3 | 147.7 | 0.0001 | 87.8 | 93.5 | 0.04 |
| Yellow vegetables | 1.0 | 0.7 | 0.21 | 5.1 | 5.3 | 0.65 |
| Tomatoes | 71.3 | 70.5 | 0.86 | 18.5 | 21.0 | 0.0002 |
| Tofu | 29.2 | 30.6 | 0.45 | 9.2 | 8.5 | 0.38 |
| Soy milk | 50.9 | 46.2 | 0.42 | 68.7 | 67.0 | 0.79 |
| Cow milk | 60.5 | 41.8 | 0.02 | 30.9 | 21.1 | 0.04 |
| Cooking oils | 18.6 | 17.5 | 0.048 | 17.6 | 17.6 | 0.95 |

suggested that endogenous oestrogens excreted via the bile are more readily metabolised and reabsorbed from the gut when the lumen contains little or no fibre. Thus, a woman on a low-fibre diet would experience greater exposure to endogenous oestrogen than a woman on a high-fibre diet (Sharpe and Skakkabaek, 1993), thereby possibly resulting in a higher breast cancer risk (Henderson *et al.*, 1991). Vitamin C and carotene are antioxidants that have been shown to protect against cancer in animals and humans, although information specific for breast cancer is relatively scant (Hunter and Willett, 1993). It is also possible that other ingredients present in Chinese green vegetables are responsible for the observed protective effect of this food group against breast cancer development.

**Acknowledgements**
We thank Kazuko Arakawa for her assistance in data analysis. This study was supported by Grant R35 CA53890 from the United States National Cancer Institute and Grant SIG-2A from the American Cancer Society.

**Appendix**
The 63 food items listed in the questionnaire used in Shanghai are: (1) rice, (2) noodles, (3) mantou (steamed bun), (4) fat/lean pork, (5) fatty pork, (6) lean pork, (7) pork chop, (8) pork spare ribs, (9) pig trotters, (10) salted pork, (11) pork liver, (12) other pig organ meats, (13) beef, (14) mutton, (15) chicken, (16) duck, (17) sausage, (18) eggs, (19) vegetable oils (rapeseed, soybean, sesame, etc.), (20) lard, (21) fresh fish, (22) fresh shrimp, (23) crab, (24) eel, (25) salted or dried fish, (26) fresh cow milk, (27) soy milk, (28) cow milk powder, (29) cakes, (30) candies, (31) sugar, (32) ice cream, (33) ice milk bars, (34) tofu bought in government stores, (35) tofu bought in private markets, (36) vegetarian chicken (a soy bean product), (37) wheat gluten, (38) dried beans or peas, (39) peanuts, (40) bokchoi, (41) spinach, (42) Chinese chives, (43) cabbage, (44) Chinese cabbage, (45) cauliflower, (46) Chinese celery, (47) bean sprouts, (48) eggplant, (49) wild rice stem, (50) lentil beans with pod, (51) string beans, (52) asparagus, (53) wax gourd, (54) cucumber, (55) carrots, (56) white radish, (57) mushrooms, (58) red and green sweet peppers, (59) tomatoes, (60) apples, (61) pears, (62) oranges and tangerines and (63) watermelon.

The 68 food items listed in the questionnaire used in Tianjin are: (1) wheat noodles, (2) rice, (3) corn noodles, (4) peanut oil, (5) rapeseed oil, (6) soybean oil, (7) sesame oil, (8) pork, (9) mutton, (10) beef, (11) chicken, (12) duck, (13) pig organ meats, (14) sausage/bacon, (15) smoked fish, (16) fresh fish, (17) salted fish, (18) dried shrimp, (19) fresh shrimp, (20) crab, (21) fresh eggs, (22) salted eggs, (23) preserved duck eggs, (24) milk, (25) tofu, (26) smelly soybean curd, (27) soy milk, (28) Chinese donut, (29) dried Chinese cabbage, (30) salted Chinese cabbage, (31) pickled Chinese cabbage, (32) seaweed, (33) kelp, (34) mushrooms, (35) peanuts, (36) Chinese cabbage, (37) bokchoi, (38) cabbage, (39) eggplant, (40) cucumber, (41) spinach, (42) string beans, (43) rape, (44) white potatoes, (45) sweet potatoes, (46) carrots, (47) white radish, (48) tomatoes, (49) Chinese celery, (50) cauliflower, (51) kohlrabi, (52) Chinese chives, (53) fennel, (54) asparagus, (55) lotus roots, (56) red and green sweet peppers, (57) wax gourd, (58) pumpkin, (59) white onion, (60) apples, (61) pears, (62) watermelon, (63) banana, (64) persimmon, (65) peach, (66) grapes, (67) jujube and (68) oranges and tangerines.

**References**

BRESLOW NE AND DAY NE. (1980). *Statistical Methods in Cancer Research*, Vol. 1, *The Analysis of Case-Control Studies*. IARC Scientific Publications No. 32. International Agency for Research on Cancer: Lyon.

GRAHAM S, ZIELEZNY M, MARSHALL J, PRIORE R, FREUDENHEIM J, BRASURE J, HAUGHEY B, NASCA P AND ZDEB M. (1992). Diet in the epidemiology of postmenopausal breast cancer in the New York State Cohort. *Am. J. Epidemiol.*, **136**, 1327-1337.

HENDERSON BE, ROSS RK AND PIKE MC. (1991). Toward the primary prevention of cancer. *Science*, **254**, 1131-1138.

HOWE GR. (1989). Re: 'Total energy intake: implications for epidemiologic analyses'. *Am. J. Epidemiol.*, **129**, 1314-1315.

HOWE GR. (1992). High-fat diets and breast cancer risk. The epidemiologic evidence. *JAMA*, **268**, 2080-2081.

HOWE GR, HIROHATA T, HISLOP TG, ISCOVICH JM, YUAN J-M, KATSOUYANNI K, LUBIN F, MARUBINI E, MODAN B, ROHAN T, TONIOLO P AND YU S. (1990). Dietary factors and risk of breast cancer: combined analysis of 12 case-control studies. *J. Natl Cancer Inst.*, **82**, 561-569.

HUNTER DJ AND WILLETT WC. (1993). Diet, body size, and breast cancer. *Epidemiol. Rev.*, **15**, 110-132.

HUNTER DJ, MANSON JE, COLDITZ GA, STAMPFER MJ, ROSNER B, HENNEKENS CH, SPEIZER FE AND WILLETT WC. (1993). A prospective study of the intake of vitamins C, E, and A and the risk of breast cancer. *N. Engl. J. Med.*, **329**, 234-240.

INSTITUTE OF NUTRITION AND FOOD HYGIENE, CHINESE ACADEMY OF PREVENTIVE MEDICINE. (1980). *Chinese Food Composition Tables*. New China Press: Beijing.

INSTITUTE OF NUTRITION AND FOOD HYGIENE, CHINESE ACADEMY OF PREVENTIVE MEDICINE. (1991). *Chinese Food Composition Tables*, 2nd edn. New China Press: Beijing.

LEE HP, GOURLEY L, DUFFY SW, ESTEVE J, LEE J AND DAY NE. (1991). Dietary effects on breast cancer risk in Singapore. *Lancet*, **337**, 1197-1200.

ROHAN TE, HOWE GR, FRIEDENREICH CM, JAIN M AND MILLER AB. (1993). Dietary fiber, vitamins A, C, and E, and risk of breast cancer: a cohort study. *Cancer Causes Control*, **4**, 29-37.

SHARPE RM AND SKAKKEBAEK NE. (1993). Are oestrogens involved in falling sperm counts and disorders of the male reproductive tract? *Lancet*, **341**, 1392-1395.

WANG Q-S, ROSS RK, YU MC, NING J-P, HENDERSON BE AND KIMM HT. (1992). A case-control study of breast cancer in Tianjin, China. *Cancer Epidemiol. Biomarkers Prev.*, **1**, 435-439.

WHITTEMORE AS, WU-WILLIAMS AH, LEE M, ZHENG S, GALLAGHER RP, JIAO D, ZHOU L, WANG X, CHEN K, JUNG D, TEH C-Z, LING C, XU JY, PAFFENBARGER RS AND HENDERSON BE. (1990). Diet, physical activity, and colorectal cancer among Chinese in North America and China. *J. Natl Cancer Inst.*, **82**, 915-926.

WILLETT WC, HUNTER DJ, STAMPFER MJ, COLDITZ G, MANSON JE, SPIEGELMAN D, ROSNER B, HENNEKENS CH AND SPEIZER FE. (1992). Dietary fat and fiber in relation to risk of breast cancer. An 8-year follow-up. *JAMA*, **268**, 2037-2044.

YUAN J-M, YU MC, ROSS RK, GAO Y-T AND HENDERSON BE. (1988). Risk factors for breast cancer in Chinese women in Shanghai. *Cancer Res.*, **48**, 1949-1953.

ZARIDZE DG, LIFANOVA Y, MAXIMOVITCH D, DAY NE AND DUFFY SW. (1991). Diet, alcohol consumption and reproductive factors in a case-control study of breast cancer in Moscow. *Int. J. Cancer*, **48**, 493-501.


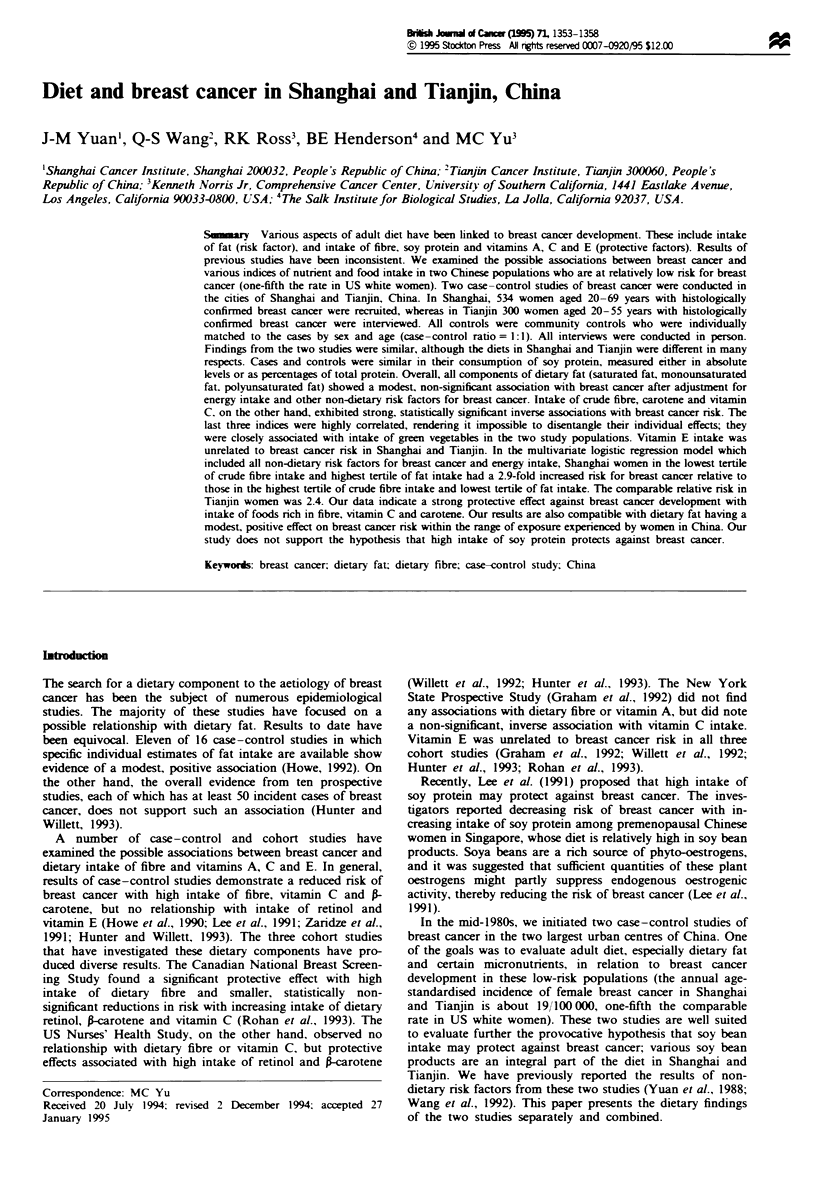

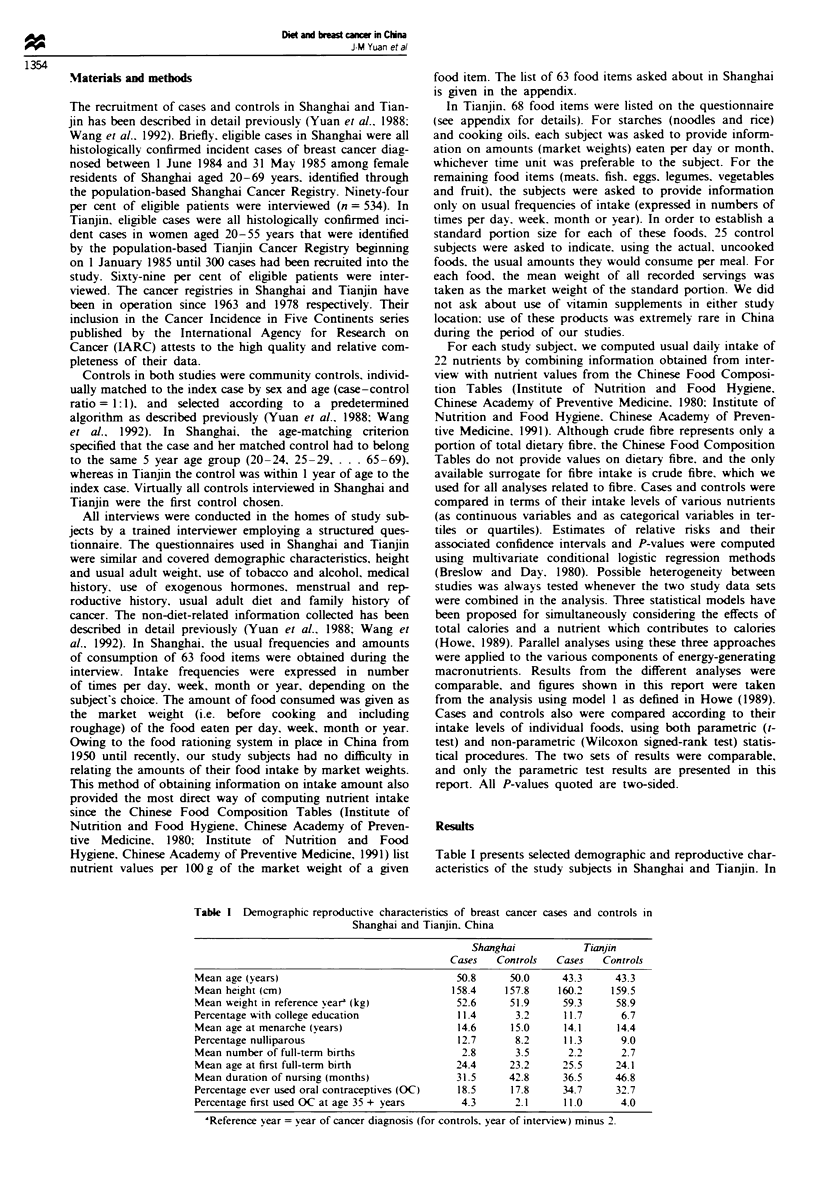

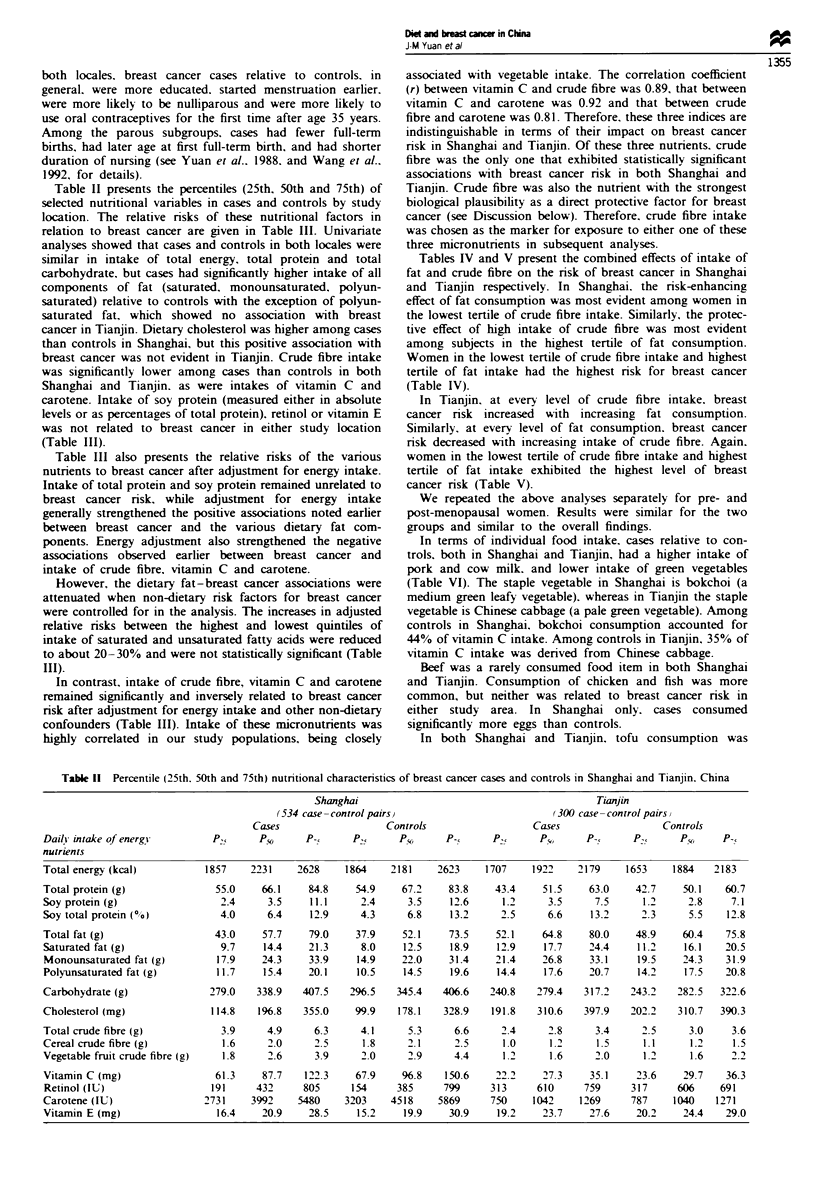

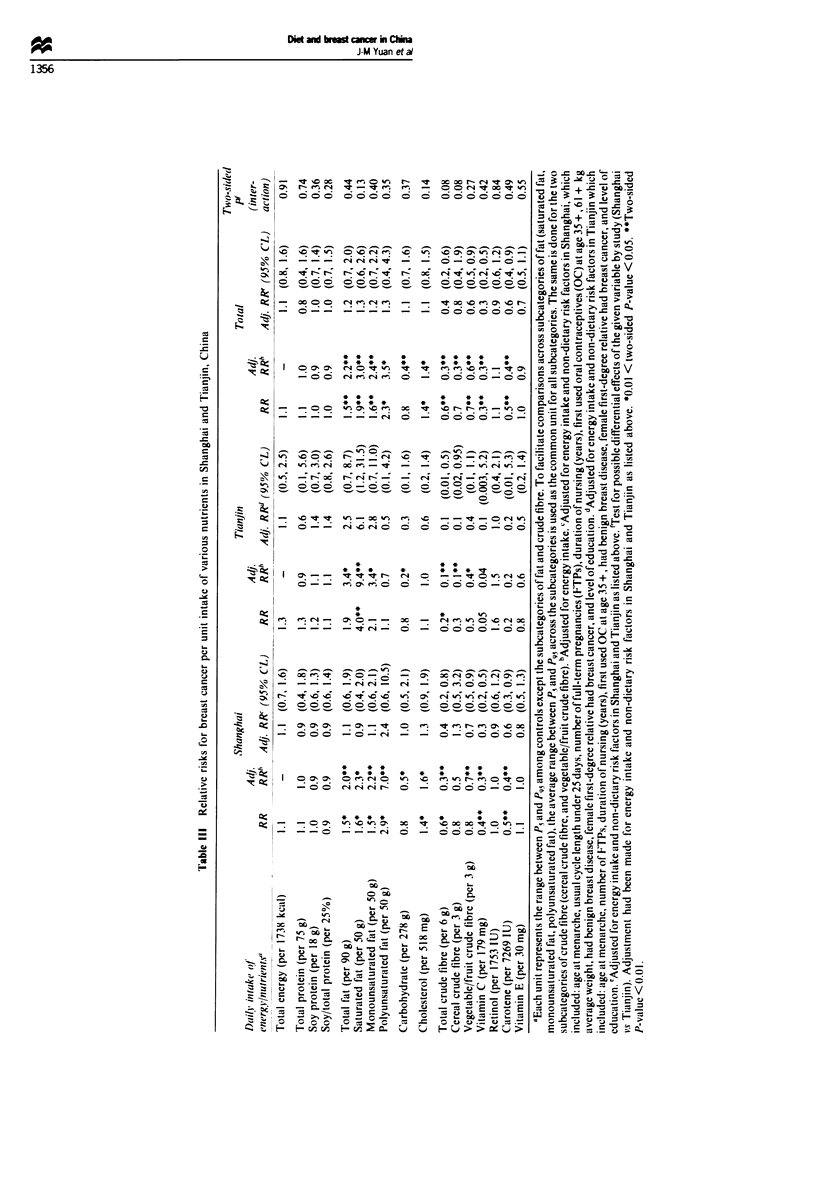

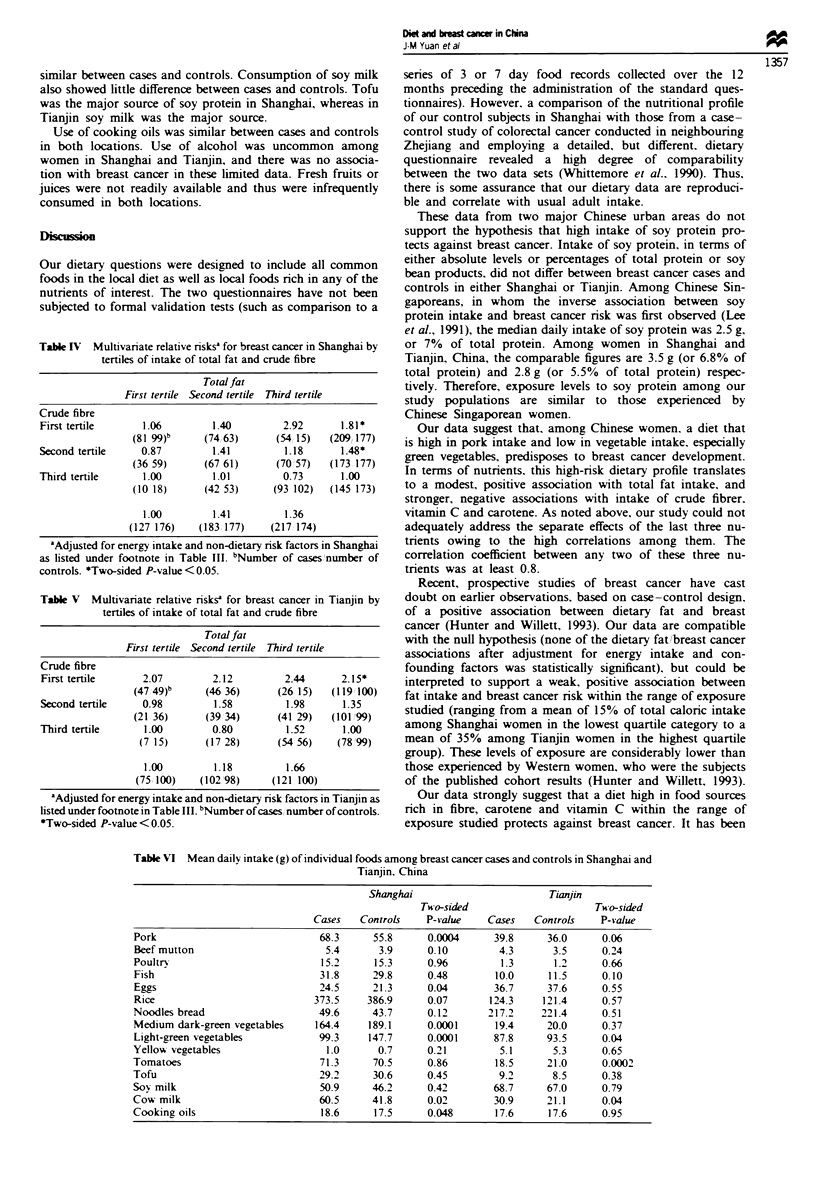

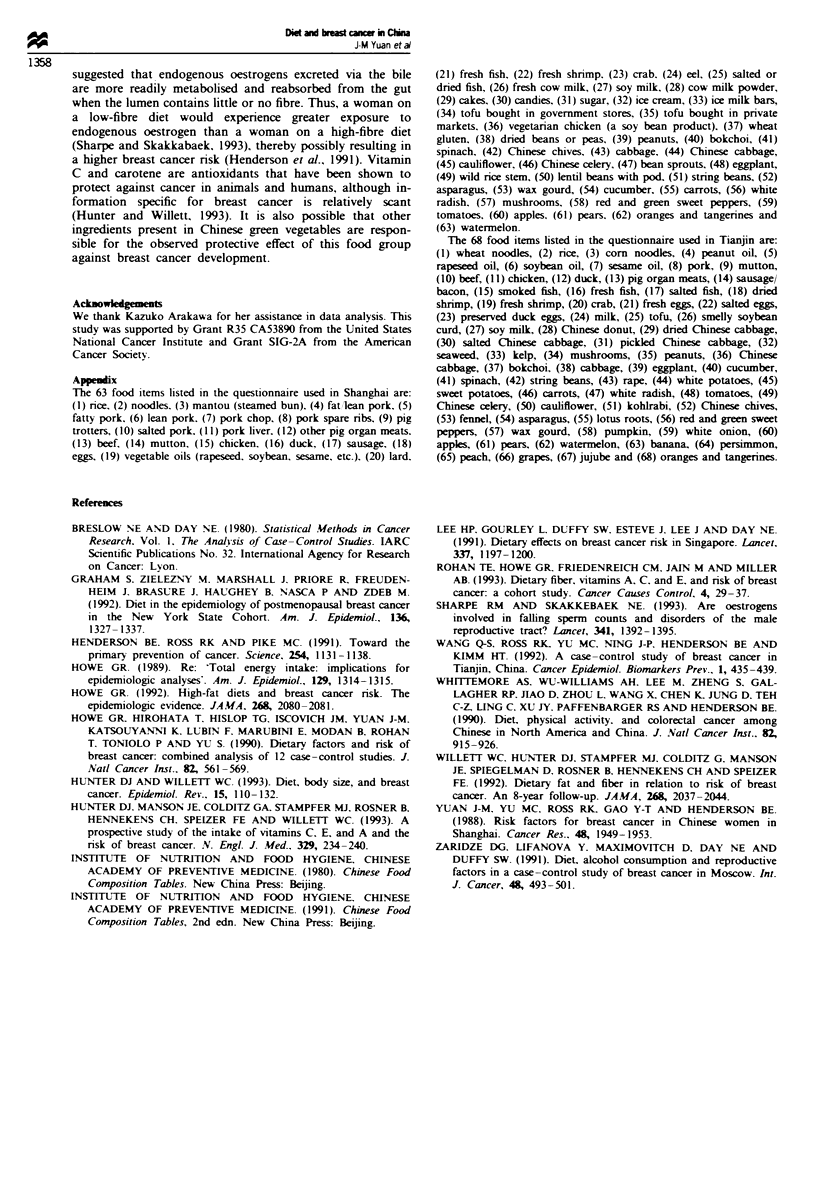


## References

[OCR_00850] Graham S., Zielezny M., Marshall J., Priore R., Freudenheim J., Brasure J., Haughey B., Nasca P., Zdeb M. (1992). Diet in the epidemiology of postmenopausal breast cancer in the New York State Cohort.. Am J Epidemiol.

[OCR_00856] Henderson B. E., Ross R. K., Pike M. C. (1991). Toward the primary prevention of cancer.. Science.

[OCR_00864] Howe G. R. (1992). High-fat diets and breast cancer risk. The epidemiologic evidence.. JAMA.

[OCR_00869] Howe G. R., Hirohata T., Hislop T. G., Iscovich J. M., Yuan J. M., Katsouyanni K., Lubin F., Marubini E., Modan B., Rohan T. (1990). Dietary factors and risk of breast cancer: combined analysis of 12 case-control studies.. J Natl Cancer Inst.

[OCR_00877] Hunter D. J., Manson J. E., Colditz G. A., Stampfer M. J., Rosner B., Hennekens C. H., Speizer F. E., Willett W. C. (1993). A prospective study of the intake of vitamins C, E, and A and the risk of breast cancer.. N Engl J Med.

[OCR_00875] Hunter D. J., Willett W. C. (1993). Diet, body size, and breast cancer.. Epidemiol Rev.

[OCR_00895] Lee H. P., Gourley L., Duffy S. W., Estéve J., Lee J., Day N. E. (1991). Dietary effects on breast-cancer risk in Singapore.. Lancet.

[OCR_00898] Rohan T. E., Howe G. R., Friedenreich C. M., Jain M., Miller A. B. (1993). Dietary fiber, vitamins A, C, and E, and risk of breast cancer: a cohort study.. Cancer Causes Control.

[OCR_00905] Sharpe R. M., Skakkebaek N. E. (1993). Are oestrogens involved in falling sperm counts and disorders of the male reproductive tract?. Lancet.

[OCR_00910] Wang Q. S., Ross R. K., Yu M. C., Ning J. P., Henderson B. E., Kimm H. T. (1992). A case-control study of breast cancer in Tianjin, China.. Cancer Epidemiol Biomarkers Prev.

[OCR_00912] Whittemore A. S., Wu-Williams A. H., Lee M., Zheng S., Gallagher R. P., Jiao D. A., Zhou L., Wang X. H., Chen K., Jung D. (1990). Diet, physical activity, and colorectal cancer among Chinese in North America and China.. J Natl Cancer Inst.

[OCR_00922] Willett W. C., Hunter D. J., Stampfer M. J., Colditz G., Manson J. E., Spiegelman D., Rosner B., Hennekens C. H., Speizer F. E. (1992). Dietary fat and fiber in relation to risk of breast cancer. An 8-year follow-up.. JAMA.

[OCR_00926] Yuan J. M., Yu M. C., Ross R. K., Gao Y. T., Henderson B. E. (1988). Risk factors for breast cancer in Chinese women in Shanghai.. Cancer Res.

[OCR_00931] Zaridze D., Lifanova Y., Maximovitch D., Day N. E., Duffy S. W. (1991). Diet, alcohol consumption and reproductive factors in a case-control study of breast cancer in Moscow.. Int J Cancer.

